# The role of complement in nonalcoholic fatty liver disease

**DOI:** 10.3389/fimmu.2022.1017467

**Published:** 2022-09-29

**Authors:** Zhenya Guo, Xiude Fan, Jianni Yao, Stephen Tomlinson, Guandou Yuan, Songqing He

**Affiliations:** ^1^ Division of Hepatobiliary Surgery, The First Affiliated Hospital of Guangxi Medical University, Nanning, China; ^2^ Key Laboratory of Early Prevention and Treatment for Regional High Frequency Tumor (Guangxi Medical University), Ministry of Education, Nanning, China; ^3^ Guangxi Key Laboratory of Immunology and Metabolism for Liver Diseases, Guangxi Medical University, Nanning, China; ^4^ Department of Endocrinology, Shandong Provincial Hospital Affiliated to Shandong First Medical University, Jinan, China; ^5^ Department of Microbiology and Immunology, Medical University of South Carolina, Charleston, SC, United States

**Keywords:** complement system, nonalcoholic fatty liver disease, site-targeted inhibitor, inflammation, liver

## Abstract

Nonalcoholic fatty liver disease (NAFLD) has become a leading cause of chronic liver diseases globally. NAFLD includes a range of hepatic manifestations, starting with liver steatosis and potentially evolving towards nonalcoholic steatohepatitis, cirrhosis or even hepatocellular carcinoma. Although the pathogenesis of NAFLD is incompletely understood, insulin resistance and lipid metabolism disorder are implicated. The complement system is an essential part of the immune system, but it is also involved in lipid metabolism. In particular, activation of the alternative complement pathway and the production of complement activation products such as C3a, C3adesArg (acylation stimulating protein or ASP) and C5a, are strongly associated with insulin resistance, lipid metabolism disorder, and hepatic inflammation. In this review, we briefly summarize research on the role of the complement system in NAFLD, aiming to provide a basis for the development of novel therapeutic strategies for NAFLD.

## Introduction

Nonalcoholic fatty liver disease (NAFLD) is defined as a condition of excess fatty deposition in the liver in the absence of excessive alcohol consumption. NAFLD is a common liver disorder, with a wide spectrum range from simple steatosis to nonalcoholic steatohepatitis (NASH), cirrhosis, or even hepatocellular carcinoma. Given the worldwide prevalence of around 25%, NAFLD is a significant global health burden (1, 2). The early stage of NAFLD, nonalcoholic fatty liver (NAFL), is characterized by simple hepatocyte steatosis without significant inflammation that leads to liver injury. NAFL is considered benign and reversible. However, the progressive stage, NASH, accompanied by inflammation and liver damage, may progress to cirrhosis and even liver cancer (3). The pathogenesis of NAFLD has often been explained in terms of a “double-hit’’ hypothesis. The “first hit” is fat deposition in hepatocytes, which originates from an imbalance between lipid in (i.e., fatty acid uptake and lipogenesis) and lipid out (i.e., fatty acid oxidation and secretion in the form of very low-density lipoprotein) (4). The “second hit” refers to oxidative stress resulting from lipid peroxidation, leading to liver inflammation and later fibrosis (5, 6). As research has progressed, a “multiple hit” hypothesis was proposed to better describe the multiple pathways that contribute to NASH (7). According to the “multiple hit” hypothesis, multiple insults act together to induce NAFLD. However, no matter which hypothesis is applied, insulin resistance and lipid metabolism disorder are considered key mechanisms contributing to NAFLD. In this review, we discuss the role of the complement system in the progression of NAFLD, aiming to elucidate the pathogenesis of NAFLD and to provide a premise for novel potential therapeutic strategies.

## Overview of the complement system

**Figure 1:**
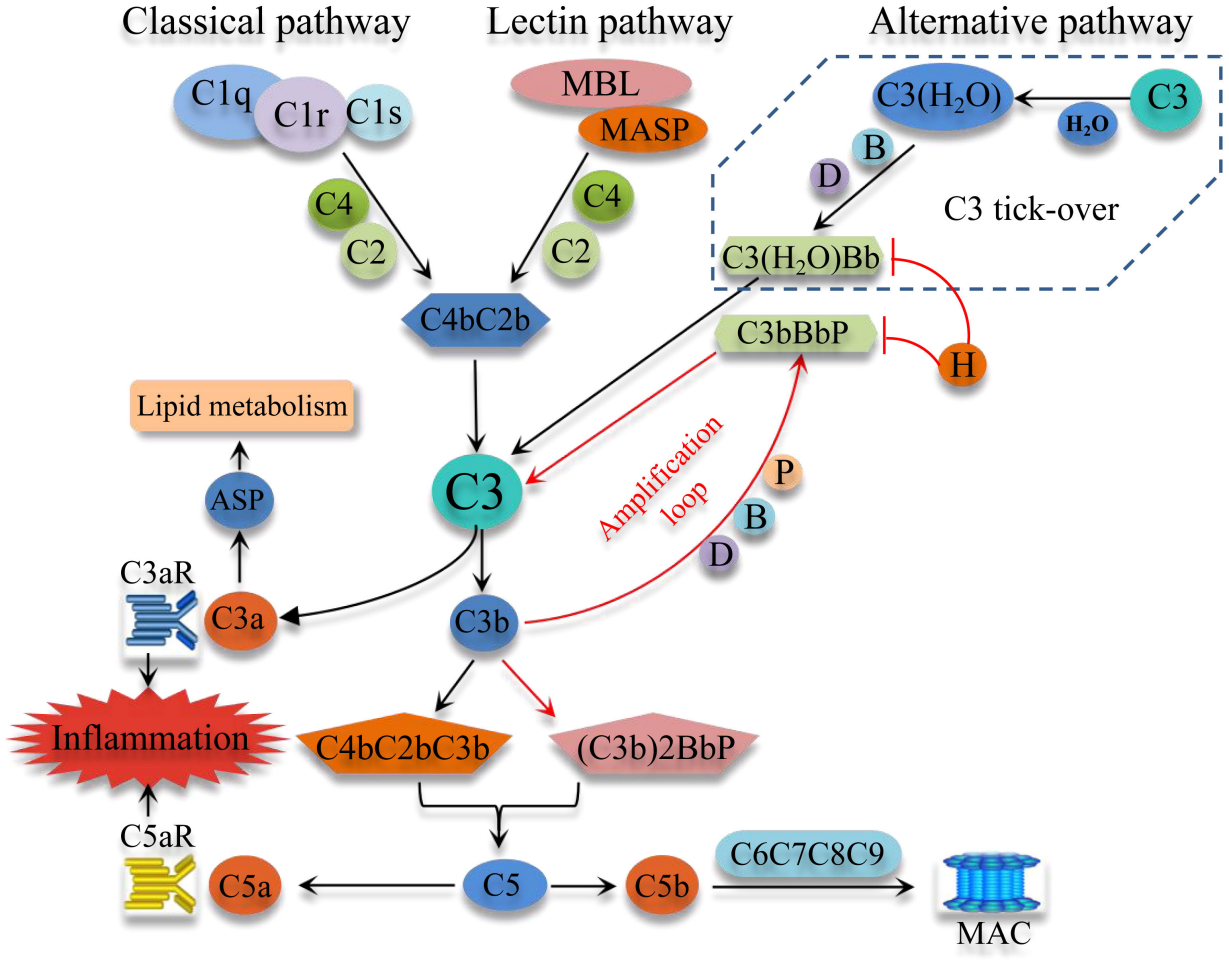
Schematic overview of the complement system activation. Complement system can be commonly activated through three pathways, the classical pathway, the Lectin pathway, and the alternative pathway (AP). Importantly, the AP can be initiated spontaneously by the “C3 tick-over”, which keeps AP idling and has the ability of fast-response through an amplification loop. These three pathways converge at C3 and produce C3 convertase and C5 convertase, which cleave C3 and C5, thereby generating C3a and C5a, important potent pro-inflammatory molecules. The activation of C5 eventually activates the downstream C6, C7, C8 and C9 to form the MAC. MBL, mannose-binding lectin; MASP, mannose-binding lectin-associated serine protease; MAC, membrane attack complex; ASP, acylation stimulating protein; B, factor B; D, factor D; H, factor H; P, properdin.

The complement system, an important component of immunity, consists of blood proteins, receptors, and soluble and membrane-bound regulatory factors, with the soluble components being mainly synthesized by the liver. The complement system can be activated through three pathways, namely the classical pathway (CP), the alternative pathway (AP), and the lectin pathway (LP) (refer to **Figure 1**). The CP is commonly activated by C1q recognition of immune complexes, leading to the activation of the serine proteases C1r and C1s. These proteases then activate C4 and C2, leading to the formation of the CP C3 convertase (C4bC2b). The LP is initiated by pattern recognition molecules (mannose binding lectin, ficolins) binding to certain carbohydrates and injured cells (pathogen-associated molecular patterns), leading to activation of LP-associated serine proteases and the formation of the CP C3 convertase. The AP, on the other hand, can be initiated spontaneously through a process termed “C3 tick-over”. The “C3 tick-over“ theory was proposed to explain the presence of the initial C3b molecules in human plasma under normal conditions. According to this theory, C3 is hydrolyzed by nucleophilic attack of H2O to form C3(H2O). Then C3(H2O) binds factor B, which is in turn cleaved by factor D, to generate the initial C3 convertase C3(H2O)Bb. The spontaneous “C3 tick-over” reaction is at a slow but constant rate in the plasm (8). The resulting C3(H2O)Bb cleaves C3 into C3b to generate the AP C3 convertase C3bBb in an amplification loop. The molecule properdin binds to and stabilizes C3bBb, which can bind an additional C3b molecule to form the C5 convertase, (C3b)2BbP. The AP also functions as an amplification loop for the CP and LP. Factor H is a critical negative regulator of the AP, which inhibits formation of, accelerates the decay of, and inactivates C3(H2O)Bb and C3bBb. The absence of factor H results in uncontrolled amplification of the AP (9). Interestingly, though the convertase activity of C3(H2O)Bb is much lower than that of C3bBb, C3(H2O)Bb is more resistant to the inactivation of factor H (8).

All three pathways converge at C3 and produce C3 and C5 convertases, which respectively cleave the central components C3 and C5 to generate the anaphylatoxins C3a and C5a. These bioactive peptides have diverse effects and play important roles in inflammation and immunity. The cleavage of C5 and generation of C5b results in the recruitment and self-assembly of C6, C7, C8 and C9 to form the cytolytic membrane attack complex (MAC).

## The complement is involved in NAFLD

The complement system was traditionally considered a component of innate immunity and inflammation, although it is now recognized as a central component of both innate and adaptive immunity. In recent years, an increasing number of studies have shown that the complement system also plays key roles in NAFLD. A Chinese cross-sectional study indicated that serum C3 levels positively correlated with the prevalence and severity of NAFLD (10); the data showed that people with higher serum C3 levels were more prone to have NAFLD. Moreover, NAFLD patients with higher serum C3 levels were more likely to have increased liver enzyme levels, which is considered a more serious form of NAFLD (11). A Turkish study came to a similar conclusion that patients with NAFLD correlate with higher serum levels of C3 and acylation stimulating protein (ASP), the desarginated form of C3a (C3adesArg). In addition, a Dutch cross-sectional study suggested that plasma C3a levels were associated with liver fat percentage (12). Histological studies provided further evidence of a link between the complement system and NAFLD. It was found that in 74% of NAFLD patients, there was deposition of activated forms of C3 and C4 in the liver tissue; in most C3-positive livers, C1q and MBL deposition was also observed, and 50% of activated C3-positive patients presented MAC formation in the liver. Interestingly, NASH is more prevalent in MAC or activated C3 positive patients (13). Moreover, in another clinical trial, it was found that AP-related C3 activation contributes to hepatic inflammation in NASH; deposition of properdin, a positive regulator of the AP, and of the C3 activation product C3c, positively correlated with liver inflammation level in NASH (14). These findings imply that the complement system can be activated through all three pathways in NASH, and that the level of complement activation is positively related to the severity of NASH. Activators of the complement system are diverse, but in NAFLD apoptotic cells may initiate complement activation. Lipid accumulation is considered the “first hit” initiating extrinsic apoptosis in NAFLD (15, 16). Both C1q and MBL can recognize apoptotic cells, thus resulting in activation of the CP and LP (17, 18). Together, the above data suggest that the complement is closely involved in the development of NAFLD.

## The complement system in insulin resistance and lipid metabolism disorder

**Figure 2:**
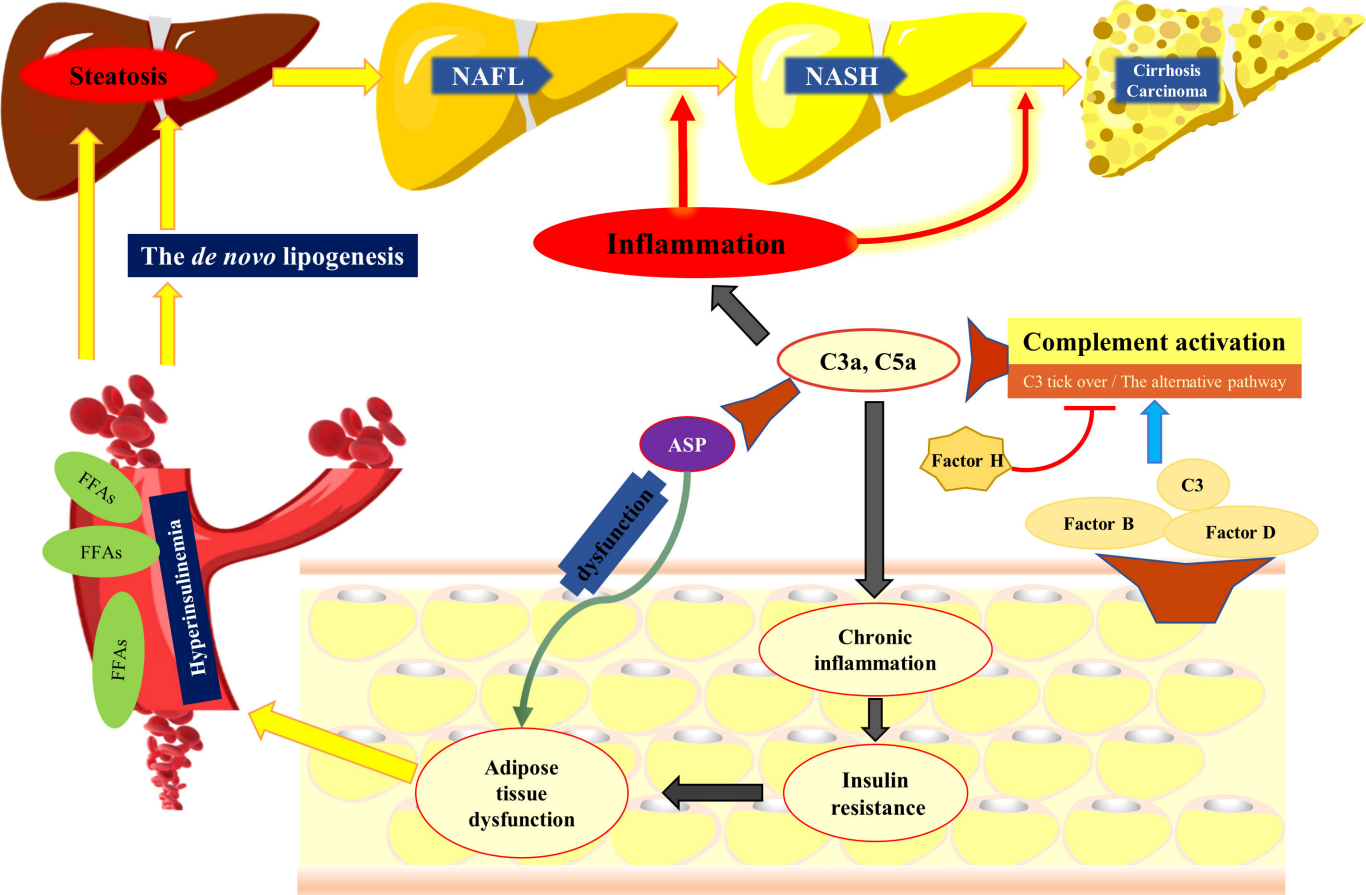
The complement system participates in the development of NAFLD. Adipose tissue can produce complement components, such as C3, factor B and factor D, which are critical for the activation of the alternative pathway. Activation of the complement system results in generation of C3a and C5a, both of which have potent pro-inflammatory activity and contribute to insulin resistance. ASP, the desarginated form of C3a, has insulin like effects, which stimulates the synthesis of triacylglycerol by transporting FFASs into adipocytes. However, when the dysfunction of ASP and insulin resistance compromises the ability of adipocytes to store fat, FFAs are released into the circulation, available for uptake by the liver. In addition, hyperinsulinemia promotes the *de novo* lipogenesis and suppresses the b-oxidation of FFAs, contributing to lipid accumulation in the liver. The complement system is also involved in NASH by regulating hepatic inflammatory response. NAFL, nonalcoholic fatty liver; NASH, nonalcoholic steatohepatitis, FFAs, free fatty acids; ASP, acylation stimulating protein.

Although the pathogenesis of NAFLD is incompletely understood, obesity, insulin resistance, and lipid metabolism disorders are implicated. Accumulating data indicate that NAFLD is a hepatic manifestation of a systemic metabolic disease rather than a confined liver disease (19). While components of the complement system are mainly synthesized by the liver, adipose tissues can also produce some complement components, such as C3, factor B and factor D (20, 21). In particular, adipose tissue is the main source of the AP protein, factor D (22). A clinical trial showed that a positive correlation between serum C3, C4, and ASP levels with obesity. Specifically, the level of C3 increased proportionally to the total amount of adipose tissue (23, 24). Moreover, increases in factor B and factor D levels were observed in obesity, while weight loss resulted in reduction of these factors. In addition, there is a positive correlation of circulating factor H concentration with obesity and insulin resistance (25). Importantly, serum C3 level is considered as a pro-inflammatory biomarker contributing to low-grade inflammation and insulin resistance in obesity (26). Under physiological conditions, the AP is activated spontaneously through the “C3 tick-over”, which requires the participation of factors B and D (27). This implies that AP activation is involved in obesity and insulin resistance. As expected, it has been shown that inhibiting the AP by factor D deficiency downregulated the expression of the genes contributing to fatty acid uptake and *de novo* lipogenesis in the liver, which eventually led to the alleviation of NAFLD (28). On the other hand, acceleration of the AP by factor H deficiency resulted in lipid accumulation and a pro-inflammatory environment in the liver (29). “C3 tick-over” also leads to the generation of low-level serum C3a, which is further degraded to ASP by serum carboxypeptidase. ASP has insulin-like effects, and stimulates the synthesis of triacylglycerols by transporting free fatty acids (FFAs) into adipocytes (24, 30). Previous studies have demonstrated that plasma ASP levels in obese individuals are significantly higher than those in nonobese people, and when obese individuals lose weight, the circulating ASP level decreases (31, 32). A study of hyperlipidemia patients showed that fasting plasma C3 and ASP levels were higher in hyperlipidemia patients than that of healthy people; oral fat loading test in healthy subjects led to a significant increase in plasma C3, while in hyperlipidemia patients, a delayed C3 response was observed (33). It was also observed that postprandial C3 increase negatively correlated with postprandial FFAs (33). Chylomicron, the form in which dietary fat is carried, can accelerate the “C3 tick-over” and activate the AP, thereby inducing the overproduction of ASP by regulating factor H (34). In addition, hydrolysis of C3 to C3(H2O) in the “C3 tick-over” could also be significantly accelerated by the interaction between C3 and a number of lipid surfaces and complexes (35). This implies that the “C3 tick-over” may be involved with lipid metabolism. Since C3 is the precursor for ASP and acts on FFA uptake by adipocytes, it is reasonable to assume that in hyperlipidemia patients, an impaired C3/ASP response may be associated with impaired FFA uptake, leading to increased plasma FFA concentration and subsequently enhanced FFA flux to the liver (**Figure 2**).

Accumulation of triglycerides (TGs) in hepatocytes is a hallmark of NAFLD (4). The liver can synthesize TGs by uptake of peripheral FFA or by producing lipids from carbohydrates (the *de novo* lipogenesis). When insulin resistance compromises the ability of adipocytes to store fat, FFAs are released into the circulation, and become available for uptake by the liver (36, 37). Accumulation of FFAs in hepatocytes enhances the synthesis of TGs, during which time the liver is also exposed to high levels of insulin (hyperinsulinemia) as well as to high levels of FFAs. The hyperinsulinemia leads to the upregulation of sterol regulatory element binding protein-1c (38), a critical transcriptional regulator of the genes involved in *de novo* lipogenesis, contributing up to 25% of total hepatic lipid stores (39). Hyperinsulinemia can suppress the β-oxidation of FFAs, contributing to lipid accumulation in the liver (40). A previous study indicated that increased C3 levels lead to higher levels of ASP in obese people, and it is conceivable that “C3 tick-over” also results in the activation of C5 and generation of C3a and C5a (23). In a high-fat diet mouse model it was shown that C5 deficiency resulted in less steatosis and lower liver inflammation, and additionally inhibited the expression of several pro-inflammatory cytokines, including IL-17 and TGF-β (41). IL-17 aggravates liver steatosis and inflammation (42), and TGF-β is related with insulin resistance (43, 44). Additionally, C3a receptor (C3aR) deficiency was protective against murine NAFLD. In this regard, C3aR is highly expressed in adipocytes and macrophages in adipose tissues (45), and C3 expression is significantly increased in adipose tissue of mice fed a high-fat diet. Interestingly, C3aR expression in the liver does not vary significantly (45). This implies that C3aR deficiency may attenuate NAFLD indirectly through affecting the adipose tissues. Importantly, the data support that the C3a/C3aR and the C5a/C5aR axes contribute to macrophage accumulation and M1 polarization in the adipose tissues of obese individuals, thereby leading to low-grade inflammation and insulin resistance (45, 46). In addition, data from rat model showed that C3aR and C5aR antagonists dramatically attenuated high-fat diet-induced obesity, insulin resistance, and adipose inflammation (47).

Taken together, the above data indicate that under physiological conditions, complement activation participates in lipid metabolism, while aberrant complement activation is involved in insulin resistance and lipid metabolism disorder which contributes to hepatocyte steatosis.

## The complement system in NAFLD progression

The involvement of complement in metabolic disorder could be regarded as its contribution to the “first hit”. Nevertheless, complement also participates in the “second hit”, that is, the progression of NAFLD. Entirely distinct from NAFL, NASH is characterized by inflammation and hepatocyte injury. However, the mechanisms driving simple steatosis to NASH are unclear. The progression of NAFLD occurs together with an aberrant activation of the immune system promoted by multiple genetic and environmental factors (48). Apart from parenchymal cells, the liver also contains a large number of complex immune cells, such as macrophages, stellate cells, and lymphoid cells. Therefore, the liver is considered not only as a metabolic organ, but also as an immune organ, which maintains homeostasis under physiological conditions. Disruption of homeostasis could be triggered by aberrant innate immune response, resulting in liver injury, fibrosis, and even carcinogenesis (49, 50). Complement, a key player in an innate immune response, can thus participate in the progression of NAFLD by modulating a variety of immune cells in the liver.

### The complement and macrophages in NAFLD

The pathogenesis of NAFLD is multifactorial, although it has been shown that an innate immune response is a key contributing factor, with macrophages playing a critical role (51). The liver harbors the largest proportion of macrophages in the body, which mainly comprise liver-resident macrophages, or Kuppfer cells (KCs), and monocyte-derived macrophages (MoMF) (52). There is abundant evidence that macrophages are involved in the development of liver steatosis, inflammation, and fibrosis in NAFLD (51, 53). Upon stimulation, KCs and MoMFs can differentiate into either pro-inflammatory so called M1-like macrophages or into anti-inflammatory M2-like macrophages. Although there is a wide spectrum of macrophage differentiation that is no longer categorized into M1 and M2, it is recognized that macrophage polarization plays a key role in the regulation of an inflammatory response. Importantly, it has been reported that KCs and MoMFs present a remarkable shift toward an M1-like phenotype in the liver of NASH mice (54), which is closely associated with the progression of NAFLD (51, 55). Activated Kupffer cells/macrophages in the liver secrete inflammatory chemokines that recruit peripheral monocytes into the liver, which further expands the inflammatory response in NAFLD (48). In addition to a pro-inflammatory effect, activated macrophages can promote liver fibrosis by regulating hepatic stellate cells (HSCs). By producing cytokines, such as TGF-β, TNF-α, and IL-1β, activated KCs promote HSC differentiation into a pro-fibrogenic phenotype, which synthesizes abundant collagen and α-smooth muscle actin, leading to liver fibrosis (56). It is well documented that complement activation products, particularly C3a and C5a, interact with a variety of complement receptors on macrophages, thereby modulating an inflammatory response. Both C3a and C5a have potent chemoattractant and pro-inflammatory properties, and their receptors are widely expressed, including on macrophages (45, 57). It has been shown that by inducing NLRP3 inflammasome activation through regulating the efflux of adenosine triphosphate, the C3a/C3aR axis contributes to IL-1β secretion in macrophages (58). C5a can also trigger NLRP3 inflammasome activation, including the release of IL-1β and TNFα release (59). In a high-fat diet mouse model, Mamane et al. (45) found that both macrophages in white adipose tissue and Kupffer cells in the liver express significant amounts of C3aR. Furthermore, C3aR deficiency improved insulin sensitivity, decreased macrophage infiltration, and inhibited the pro-inflammatory effect of M1-like macrophages. Importantly, C3aR deficiency significantly alleviated liver injury in mice fed a high-fat diet (45). Interestingly, in another study, the C5a/C5aR axis was demonstrated to also contribute to macrophage accumulation and M1-like polarization in obese white adipose tissues and thereby to insulin resistance (46). Thus, it can reasonably be speculated that the C3a/C3aR and C5a/C5aR axis may play an important role in the pathogenesis of NAFLD *via* activating Kupffer/macrophages.

### The complement and neutrophils in NAFLD

In addition to macrophages, neutrophils have also been shown to be involved in the pathogenesis of NAFLD. Several studies in humans and mice have suggested that neutrophil infiltration in the liver contributes to the progression of NAFLD. The level of circulating neutrophils and their infiltration are closely related to the severity of NAFLD (60–63). Consistent with these findings, reactive oxygen species (ROS) produced by infiltrating neutrophils contribute to the progression to NASH (64). In NASH patients, plasma myeloperoxidase (MPO) levels are higher (65), and MPO induces liver cell death and activation of HSCs, which contribute to liver fibrosis. In contrast, inhibiting neutrophil infiltration in the mouse liver can significantly reduce NAFLD progression (66). MPO deficiency alleviates liver injury by suppressing HSC activation, leading to decreased TGF-β levels and fibrosis in mice (67). C3a and C5a also have potent biological activities for neutrophils. The effect of C3a on neutrophils is somewhat unclear. While C3a is often considered pro-inflammatory and its receptor C3aR is highly expressed on neutrophils (68), emerging data show that the C3a/C3aR axis also has anti-inflammatory effects (69, 70). Nevertheless, it is clear that C5a has a pro-inflammatory effect on macrophages. The C5a/C5aR axis is closely associated with neutrophil functions such as chemotaxis and respiratory burst (71). In addition, C5a can inhibit apoptosis of neutrophils (72), lead to increased vascular permeability, and promote the expression of P-selectin, thereby contributing to the infiltration of neutrophils (73). The terminal activation product of complement, the MAC, is not only cytolytic, but at sublytic concentrations can also promote the release of chemokines for neutrophil infiltration. Thus, hepatic neutrophil infiltration is also associated with MAC deposition (13).

### The complement and T cells in NAFLD

There is emerging evidence that T cells play an important role in the pathogenesis of NASH. In basic functional terms, T cells can be divided into cytotoxic CD8 T cells, CD4 T helper (Th) cells, and CD4 regulatory T cells (Tregs) (74). CD8 T cells are increased in the livers of patients with NASH (75, 76). In a mouse model, depletion of CD8 T cells alleviated NASH (77). It has been implied that CD8 T cells trigger NASH progression through generation of pro-inflammatory cytokines and non-specific killing of hepatocytes (75, 78). Moreover, an imbalance between Th1 (which secrete pro-inflammatory cytokines) and Th2 (secrete anti-inflammatory cytokines) cells appears to contribute to the pathogenesis of NAFLD (79). Inflammatory Th1 and Th17 cells have also been reported to be increased in NASH (80, 81). In addition, the progression from NAFL to NASH is marked by an increased Th17/resting Tregs ratio in the liver (80). Complement has widely been recognized to participate in the differentiation of T cells and regulate their biological function. For example, the C3a/C3aR axis is involved in modulating Th2 cell differentiation through mediating the secretion of IL-12 by antigen-presenting cells (82). It has also been suggested that C3aR and C5aR signaling contributes to the differentiation of Th1 and Th17 cells, and C5aR signaling in dendritic cells is critical for inducing T-cell differentiation into Th17 (83).

## Conclusions and future perspective

In summary, as a participant in lipid metabolism as well as immune responses, the complement system plays an important role in multiple stages of NAFLD. Components of the AP, C3, C5, and ASP have all been shown to be involved in insulin resistance, lipid metabolism disorder, and an inflammatory response (**Figure 2**). Together, the reviewed studies suggest that the complement system may be a promising therapeutic target for treating human NAFLD. Indeed, in several pre-clinical models, intervention in the complement system showed therapeutic benefit. Nevertheless, note should be taken that the complement system is an essential component of immunity and provides an important defense against pathogens. It also has multiple important physiological and homeostatic functions such as tissue repair, immune complex catabolism, and the removal of dead and dying cells (84). Therefore, improper or nonspecific intervention in the complement system carries a risk of serious side effects, including increased risk of infection. And while these are important considerations, including for most of the greater than 80 clinical trials currently ongoing with complement inhibitors, there is also a risk-benefit consideration. A strategy that could also be considered is site-targeted complement inhibition, in which complement is inhibited only locally at sites of pathology while leaving systemic activity largely intact (83). To conclude inhibition of complement may provide a promising approach to limit the progression of NAFLD and may open new avenues of treatment.

## Author contributions

ZG and GY conceived this study. SH and GY directed the study. ZG, GY, XF, and JY performed literature search. ZG drafted the manuscript. SH, ST, XF, and GY provided critical intellectual revision. SH and GY provided financial support. All authors contributed to the article and approved the final version.

## Funding

This study was supported in part by the National Natural Science Foundation of China (No. 91949122 and No. 82160500); the 111 Project (D17011); Guangxi science and technology base and talent project (GuikeAA21220002); Guangxi Key Research and Development Plan (2018AD03001); Special project of central government guiding local science and technology development (ZY20198011); Key Laboratory Base of Liver Injury and Repair of the First Affiliated Hospital of Guangxi Medical University (YYZS2020001).

## Conflict of interest

The authors declare that the research was conducted in the absence of any commercial or financial relationships that could be construed as a potential conflict of interest.

## Publisher’s note

All claims expressed in this article are solely those of the authors and do not necessarily represent those of their affiliated organizations, or those of the publisher, the editors and the reviewers. Any product that may be evaluated in this article, or claim that may be made by its manufacturer, is not guaranteed or endorsed by the publisher.
